# Holmium laser enucleation of the prostate in the oldest-old: a comparative analysis across the extremes of age

**DOI:** 10.3389/fmed.2026.1791612

**Published:** 2026-03-13

**Authors:** Hasim Bakbak, Ansh Bhatia, Juanita Velasquez, Maggie Meyreles, Ahmad Abdelaziz, Gurpremjit Singh, Archan Khandekar, Jonathan E. Katz, Robert Marcovich, Hemendra N. Shah

**Affiliations:** 1 Desai Sethi Urology Institute, University of Miami Miller School of Medicine, Miami, FL, United States; 2 Department of Interventional Radiology, University of Miami Miller School of Medicine, Miami, FL, United States

**Keywords:** benign prostatic hyperplasia, complications, endoscopic enucleation of the prostate, enlarged prostate, HoLEP, octogenarian, oldest-old

## Abstract

**Introduction:**

Expansion of the “oldest-old” (≥85 years) has increased the prevalence of BPH and the need for surgery. Holmium laser enucleation of the prostate (HoLEP) is a size-independent alternative to TURP, yet age-specific outcomes remain understudied. We compared outcomes, complications, and recovery after HoLEP in men ≥85 years versus ≤55 years.

**Methods:**

We conducted a retrospective analysis of a prospective database of HoLEP procedures performed by a single surgeon at a tertiary academic center (2017–2025). Men aged ≥85 years constituted the study cohort and were compared with men ≤55 years. Baseline demographics, frailty, perioperative variables, complications, and functional outcomes (IPSS, Qmax, PVR) were analyzed. Statistical comparisons used *t*-tests, Wilcoxon, chi-square, Fisher exact tests.

**Results:**

Sixty-one patients were included (30 ≥ 85; 31 ≤ 55). The oldest-old exhibited significantly higher comorbidities and frailty, lower baseline hemoglobin, worse renal function, and more frequent catheter dependence, while younger men had higher BMI. Operative time, hemoglobin decline, and hospital stay were comparable. Catheterization was longer in the oldest-old. The elderly had significantly more minor complications and a nonsignificant increase in major complications. UTIs occurred only in the oldest-old. Incidental prostate cancer was more common in the ≥85 cohort. Both groups showed improvements in IPSS, Qmax, and PVR; however, younger men experienced significantly greater Qmax at 12 months.

**Conclusion:**

HoLEP is safe and effective in the oldest-old, offering durable improvement despite increased frailty and risk of complications. Although younger patients exhibited greater improvements in urinary flow, it is uncertain whether this finding reflects a causal benefit of earlier intervention, underscoring the need for prospective validation. Advanced age alone should not preclude HoLEP candidacy.

## Introduction

Rising life expectancy has expanded the “oldest-old” population, defined as age 85 years and older (1). This group represents the fastest-growing age segment in the U.S., and men ≥ 85 constitute approximately 1.43% of the U.S. male population, and is projected to triple over 40 years (2). Benign prostatic hyperplasia (BPH) affects 80–90% of men ≥ 80 and is expected to impose a growing clinical burden on aging populations (3). As lower urinary tract symptoms (LUTS) increase, the demand for surgical management among elderly men will rise.

Advanced age is associated with increased perioperative risk, attributable to multimorbidity and frailty (4, 5). Longstanding bladder outlet obstruction (BOO) may further exacerbate age-related detrusor underactivity and structural bladder remodeling, limiting recovery after deobstruction (6). As a result, elderly patients undergoing surgery for BPH exhibit higher rates of complications, prolonged hospitalization, and slower convalescence.

Transurethral resection of the prostate (TURP) has historically been regarded as the reference standard for surgical management of BPH and continues to be widely performed in octogenarians (7–9). However, its use in medically complex elderly patients is constrained by elevated perioperative morbidity, including longer operative times, higher catheter dependency, and greater bleeding risk, particularly in individuals on anticoagulation (10, 11). Holmium laser enucleation of the prostate (HoLEP), a size-independent alternative endorsed by both the AUA and EAU, offers excellent hemostasis and durable symptom relief with a favorable safety profile (7, 8, 12, 13). Prior studies have demonstrated that HoLEP maintains safety even in elderly patients with elevated ASA scores or concurrent anticoagulation (14). A prospective randomized study has shown HoLEP to be a size-independent option with a favorable safety profile defined by less bleeding, transfusions, and a significantly lower hemoglobin drop compared to bipolar TURP (15).

Despite these advantages, age-specific analyses reveal important differences in perioperative risk and functional recovery. Octogenarians undergoing HoLEP consistently demonstrate longer operative duration, increased complications, extended hospitalization, and higher transfusion and readmission rates compared with younger cohorts (8, 16). These observations mirror broader evidence that elderly undergoing BPH surgery experience higher perioperative morbidity (17). Conversely, emerging data indicate that younger men (≤55 years) experience greater functional improvements after HoLEP, even though a substantial proportion present with absolute indications such as recurrent urinary retention and catheter dependence, likely reflecting preserved detrusor contractility and a lower comorbidity burden (17–19).

Given population aging and the expansion of HoLEP across a broad age spectrum, understanding how age influences surgical outcomes is of increasing clinical significance. To address this knowledge gap, the present study directly compares perioperative outcomes, functional improvement, and complication profiles following HoLEP in men ≥85 years and men younger than 55 years, representing opposite extremes of the age continuum. These age thresholds were selected to maximize contrast in physiological reserve, detrusor function and frailty burden thus providing an extremes-of-age comparison to better delineate age-associated differences than conventional strata used in the literature. We hypothesized that younger men would exhibit superior postoperative functional recovery, whereas the oldest-old would demonstrate higher perioperative morbidity and comparably modest improvements in voiding function due to worse detrusor contractility and greater comorbidity burden.

## Methods

### Study design

We retrospectively analyzed a prospectively maintained institutional database encompassing patients who underwent HoLEP at our center between July 2017 and August 2025 by a single surgeon (HNS). Men aged ≥85 years were identified as the study cohort. For comparison, we included men ≤55 years from the same database. Patients with neurogenic bladder, urethral stricture, bladder malignancy, or a history of locally advanced prostate cancer (PCa) were excluded. Individuals undergoing HoLEP as part of multimodal management prior to radiotherapy or high-intensity focused ultrasound for organ-confined PCa were also excluded. Institutional Review Board approval was obtained before data collection and analysis (IRB #20180511).

### Surgical technique and perioperative management

Prostate volume was assessed using transabdominal or transrectal ultrasonography, CT, or MRI within 12 months prior to surgery. Patients with elevated PSA underwent further investigation with 4-Kscore testing, multiparametric MRI, and/or biopsy based on shared decision making. Preoperative anticoagulant and antiplatelet therapies were withheld in coordination with the patient’s primary care physician or cardiologist. Urine culture was obtained within two weeks of the scheduled procedure and those patients with a positive culture received culture-directed antibiotic therapy prior to surgery.

All operations were performed using an en-bloc HoLEP technique as previously described (20). Over the past 2 years we modified our surgical template with an attempt to preserve anterior fibromuscular stroma and overlying mucosa (when possible). A single high-volume attending surgeon performed or directly supervised each case, with residents and endourology fellows participating. Enucleation was carried out using a 26-French laser resectoscope (Karl Storz, Tuttlingen, Germany) and a 550-μm laser fiber connected to either a 100-W or 120-W holmium: YAG laser platform incorporating Moses pulse modulation technology (Lumenis, Santa Clara, California). Tissue morcellation was completed using the Lumenis VersaCut™ system (Lumenis, Santa Clara, California) or the Wolf Piranha morcellator (Richard Wolf, Vernon Hills, IL). At procedure completion, a 22-French Foley catheter was inserted without catheter traction.

All patients were admitted overnight for continuous bladder irrigation and routine monitoring. On postoperative day (POD) 1 a complete blood count and basic metabolic panel, was obtained followed by a voiding trial. Patients demonstrating satisfactory voiding were discharged. Those unable to void or who exhibited a post-void residual volume >150 mL were discharged with a catheter and scheduled for an outpatient repeat trial 2–7 days. Patients with chronic urinary retention and postvoid residual urine of >500 cc who were not dependent on catheter before surgery were usually discharged with indwelling foley catheter for 1–3 weeks. All prostate-directed medications were discontinued immediately after surgery.

### Postoperative follow-up

Follow-up evaluations were performed at 6 weeks, and at 3, 6, and 12 months. At each visit, functional outcomes were assessed using the International Prostate Symptom Score (IPSS), maximum urinary flow rate (Qmax), and post-void residual urine volume (PVR). PSA nadir was recorded at 3 months. Complications were systematically documented. Urinary incontinence was defined as any involuntary leakage requiring pad use at any postoperative time point.

### Data collection

Data extracted included baseline demographics, comorbid conditions, preoperative anticoagulant or antiplatelet use, prostate volume, PSA level, serum creatinine, and functional parameters (IPSS, Qmax, PVR). Frailty was quantified using the 11-item modified frailty index (mFI-11), derived from the Canadian Study of Health and Aging Frailty Index, and validated across various surgical populations. In accordance with established thresholds, frailty was defined as mFI-11 ≥ 0.36. Surgical indications and intraoperative variables, including operative duration (defined as total time spent in the operating room), intraoperative complications, and weight of enucleated tissue were recorded. Postoperative outcomes included hemoglobin decrement, serum creatinine on POD 1, transfusion requirements, and length of hospitalization. All retrieved prostate tissue underwent histopathological assessment, and incidental prostate cancer (iPCa) was documented.

### Statistical analysis

Continuous variables were summarized as means with standard deviations or as medians with interquartile ranges, based on data distribution, and were compared using independent-samples *t*-tests or Wilcoxon signed rank tests as appropriate. Categorical variables were compared using chi-square or Fisher exact tests as appropriate. A two-sided *p*-value < 0.05 was considered statistically significant. Statistical analyses were performed using R version 4.3.1 (R Foundation for Statistical Computing, Vienna, Austria; 2025). Missing data were addressed through available-case analysis, with denominators reported for each outcome.

## Results

A total of 857 patients underwent HoLEP during the study period, of whom 61 met inclusion criteria, comprising 30 men aged ≥85 years and 31 men aged ≤55 years. Younger patients exhibited significantly higher BMI and obesity (BMI > 30 kg/m^2^), whereas the oldest-old demonstrated a greater comorbidity burden, reflected by higher rates of dyslipidemia and a substantially larger proportion of frail individuals based on mFI-11 criteria (Table 1). Among the individual mFI components, hypertension, peripheral vascular disease, heart failure, and a history of TIA or CVA were significantly more common in the ≥85-year cohort. COPD was observed exclusively in the oldest-old, though the difference did not reach statistical significance. No significant differences were identified across the remaining frailty items. The study group had significantly lower baseline hemoglobin and substantially worse renal function. Median preoperative eGFR was 60 vs. 79 mL/min/1.73 m^2^, and chronic kidney disease (CKD, ≥grade 3a) was present in 48.0% of men ≥85 years compared to 3.7% of younger men. High baseline PVR (≥300 mL) was more prevalent in the oldest-old but was not statistically different between groups. Catheter dependence was more common among the ≥85-year cohort, whereas the proportion performing clean intermittent catheterization (CIC) was similar. Pre-HoLEP emergency department (ED) visits within the preceding 90 days did not differ significantly.

**Table 1:** Patient demographics and baseline characteristics.

Variable	HOLEP ≥85 (*N* = 30)	HOLEP ≤55 (*N* = 31)	*p* value
Age (years)	88.4 ± 3	52.5 ± 3.2	<0.001
BMI (kg/m^2^)	24.6 ± 3.0	28.3 ± 4.3	**<0.001**
Patients with BMI > 30	1/30 (3.3%)	10/31 (32.2%)	**0.006**
Dyslipidemia	18/30 (60%)	4/31 (12.9%)	**<0.001**
Hemoglobin (g/dl)	12.5 [11.47–12.95]	14.9 [14.15–15.55]	**<0.001**
Creatinine (mg/dl)	1.1 [1.0–1.4]	1 [0.9–1.1]	0.73
Baseline eGFR (mL/min/1.73 m^2^)	60 [43–64]	79 [75–84]	**<0.001**
Baseline CKD (≥Grade 3a)	12/25 (48%)	1/27 (3.7%)	**<0.001**
PVR ≥ 300 ml	4/23 (17.4%)	4/26 (15.4%)	1
Catheter dependent-indwelling	21/30 (70%)	10/31 (32.2%)	**0.005**
Performing CIC	3/30 (10%)	1/31 (3.2%)	0.35
Pre-HoLEP ED visit (−90 days)	7/30 (23.3%)	6/31 (19.3%)	0.76
N of frail patients (mfi-11 ≥ 0.36)	11/30 (36.6%)	2/31 (6.5%)	**0.01**
mFI components, *n* (%)			
Functional dependence	2/30 (6.6%)	1/31 (3.2%)	0.61
Diabetes mellitus	11/30 (36.6%)	5/31 (16.1%)	0.08
COPD	2/30 (6.6%)	0	0.23
Congestive heart failure	4/30 (13.3%)	0	**0.05**
Myocardial infarction	9/30 (30%)	7/31 (22.5%)	0.57
PCI/cardiac surgery/angina	4/30 (13.3%)	2/31 (6.4%)	0.42
Hypertension	26/30 (86.6%)	15/31 (48.3%)	**0.002**
Peripheral vascular disease	4/30 (13.3%)	0	**0.05**
Impaired sensorium	1/30 (3.3%)	0	0.49
TIA or CVA	7/30 (23.3%)	1/31 (3.2%)	**0.03**
Anticoagulant/antiplatelet use	11/30 (36.6%)	6/31 (19.3%)	0.16
mFI score distribution, *n* (%)			
0	2/30 (6.6%)	13/31 (41.9%)	NA
1	9/30 (30%)	9/31 (29%)	NA
2	5/30 (16.6%)	2/31 (6.4%)	NA
3	3/30 (10%)	5/31 (16.1%)	NA
4	6/30 (20%)	1/31 (3.2%)	NA
5	1/30 (3.3%)	1/31 (3.2%)	NA
6	3/30 (10%)	0	NA
7–11	0	0	NA
Prostate related parameters			
*N* with prior BPO surgery	4 (13.3%)	1 (3.2%)	0.22
Detail of prior BPO surgery	2-TURP 2-PAE 1-PVP	1-PAE	NA
Prostate size (cc)	120 ± 88	100 ± 68	0.32
PSA (ng/ml)	4.9 ± 5.4	5.9 ± 5.5	0.49
Indication of BPO surgery			
Recurrent urinary retention	18/30 (60.0%)	20/31 (64.5%)	0.79
Recurrent gross hematuria	4/30 (13.3%)	4/31 (12.9%)	1
Recurrent UTI	2/30 (6.6%)	2/31 (6.4%)	1
Bothersome LUTS	17/30 (56.6%)	21/31 (67.7%)	0.43

Data are presented as mean ± SD, median [IQR], or *n*/*N* (%), as appropriate. BMI, body mass index; CKD, chronic kidney disease (stage ≥3a, estimated glomerular filtration rate [eGFR] < 60 mL/min/1.73 m^2^); PVR, post-void residual; CIC, clean intermittent catheterization; ED, emergency department; mFI-11, 11-item modified frailty index (frailty defined as mFI-11 ≥ 0.36); COPD, chronic obstructive pulmonary disease; CHF, congestive heart failure; PCI, percutaneous coronary intervention; TIA, transient ischemic attack; CVA, cerebrovascular accident; BPO, benign prostatic obstruction; LUTS, lower urinary tract symptoms; PSA, prostate-specific antigen; TURP, transurethral resection of the prostate; PAE, prostatic artery embolization; PVP, photoselective vaporization of the prostate. “NA” indicates *p* values were not calculated for descriptive distribution rows.

Boldface indicates statistical significance (*p* < 0.05).

Perioperative outcomes were similar, with no statistically significant differences in operative time, hemoglobin decline, or length of stay. No intraoperative complications occurred in either cohort. POD-1 creatinine demonstrated a nonsignificant trend toward a greater rise in older patients. Duration of postoperative catheterization differed, with both groups having a median of 1 day but a significantly broader range in the ≥85-year cohort (*p* = 0.007). Three patients in the ≥85-year cohort required postoperative ICU monitoring, whereas none of the younger patients did. ED visits within 90 days after HoLEP were similar between groups. Two patients in the study group were discharged to a rehabilitation facility, whereas all patients in the comparison group were discharged directly home. iPCa was numerically higher among the oldest-old. In the study group, two patients had known grade group 1 (GG1) disease prior to surgery which none were found to have iPCa during histopathological exam of the removed tissue. Among six patients from the elderly group who were incidentally diagnosed with prostate cancer after surgery, none had known PCa and no prior biopsy. In the comparison group, three patients had known GG1 disease prior to surgery and only one was found to have iPCa during histopathological exam of the removed tissue. Among four patients who were incidentally diagnosed with PCa after surgery, two had prior biopsies and one had prior GG1 disease.

Both age groups showed improvements in IPSS, Qmax, and PVR from baseline. Despite higher baseline PVR in ≥85 group, they experienced similar reduction in PVR as younger men. Younger patients, however, achieved greater improvement in Qmax at each follow-up time frame, reaching statistical significance at 12 months, however there was significant attrition observed at 6- and 12- month follow-up particularly among the oldest-old (Table 2).

**Table 2:** Intraoperative, postoperative, and follow-up parameters.

Variable	HOLEP ≥85		HOLEP ≤55		*p* value
Intraoperative outcomes					
Operative time (minutes)	150.4 ± 71.5		155.5 ± 69.1		0.83
Resected prostate volume (grams)	80.3 ± 71.2		70.5 ± 64.4		0.36
Postoperative outcomes					
Creatinine POD 1 (mg/dl)	1.2 ± 0.6		0.98 ± 0.2		0.06
Hgb Drop POD 1 (g/dl)	2.3 ± 2.9		1.9 ± 0.9		0.42
Duration of catheter (days)	1 [1–7]		1 [1–1]		**0.007**
Length of stay (days)	1 [1–1]		1 [1–1]		0.18
Need for ICU monitoring	3/30 (10%)		0/31 (0%)		0.11
ED visit (+90 days)	3/30 (10%)		4/31 (12.9%)		1
Incidental prostate cancer	6/30 (20%)		4/31 (12.9%)		NA
PSA at 3 months (ng/ml)	0.8 ± 1.2		0.5 ± 0.45		0.24
Functional outcomes					
IPSS	*N*	Median (IQR)	*N*	Median (IQR)	
Baseline	22	15 [10–29]	28	19 [14–24]	0.4
3 Months	14	2 [0–8]	22	2 [1–4]	1
6 Months	7	3 [2–5]	10	0 [0–4]	0.33
12 Months	6	5 [2–7]	9	0 [0–3]	0.08
Qmax (ml/s)	*N*	Median (IQR)	*N*	Median (IQR)	
Baseline	9	8.0 [3.3–9.6]	21	6.8 [4.8–11.9]	0.5
3 Months	15	11.1 [8.1–15.2]	22	21.9 [9.3–27.0]	0.07
6 Months	11	16.0 [11.1–20.6]	11	18.4 [17–25.2]	0.11
12 Months	6	8.6 [6.2–9.9]	14	20.2 [15–29.6]	**0.002**
PVR (ml)	*N*	Median (IQR)	*N*	Median (IQR)	
Baseline	23	210 (80–297)	26	129 (39–207)	0.14
3 Months	19	9 (0–41)	22	22 (1–51)	0.54
6 Months	14	6 (2–68)	11	21 (2–96)	0.64
12 Months	9	16 (0–55)	16	17 (3–49)	0.73
Complications					
1. Major CD ≥ 3	3/30 (10%)		1/31 (3.2%)		0.35
1a. Bladder neck contracture	2/30 (6.6%)		0/31 (0%)		0.24
1.b Urethral stricture	0/30 (0%)		1/31 (3.2%)		1
2. Minor CD ≤ 2	18/30 (60%)		11/31 (35.4%)		**0.04**
2a. Transfusion	3/30 (10%)		0/31 (0%)		0.11
2b. Recatheterization on POD 1	3/30 (10%)		3/31 (9.6%)		1
2c. Gross hematuria	2/30 (6.6%)		2/31 (6.4%)		1
2d. UTI requiring antibiotics	4/30 (13.3%)		0/31 (0%)		**0.05**
2e. Transient urinary incontinence (6 weeks)	16/30 (53.3%)		10/31 (32.2%)		0.12
2 f. Incontinence at 1 year	2/27 (7.4%)		2/29 (6.9%)		1

Data are presented as mean ± SD, median [IQR], or n/N (%), as appropriate. POD, postoperative day; Hgb, hemoglobin; ED, emergency department; ICU, intensive care unit; IPSS, International Prostate Symptom Score; Qmax, maximum urinary flow rate; PVR, post-void residual; UTI, urinary tract infection; CD, Clavien–Dindo complication grade (major defined as CD ≥3; minor defined as CD ≤2). PSA, prostate-specific antigen. Bladder neck contracture and urethral stricture were considered major complications. “NA” indicates *p* values were not calculated (e.g., descriptive rows or insufficient numbers for reliable testing). Boldface indicates statistical significance (*p* < 0.05).

Overall complication rates were higher in the ≥85-year cohort. Minor complications (Clavien–Dindo ≤2) were more frequent in the oldest-old group than in younger men (60.0% vs. 35.4%), and this difference was statistically significant; major complications (Clavien–Dindo ≥3) also occurred more often in the oldest-old (10.0% vs. 3.2%) but did not reach statistical significance. Three patients in the ≥85-year cohort required blood transfusion. Of these patients, two had documented preoperative anemia, and one transfusion was attributed to postoperative hematuria. Three patients among the elderly required postoperative ICU monitoring. Consistent with other perioperative complications, transient urinary incontinence was more frequent in the oldest-old, although this difference did not reach statistical significance. Persistent incontinence at 12 months was comparable between groups. UTIs requiring antibiotics occurred only in the ≥85 cohort (13.3% vs. 0%, *p* = 0.05) (Table 2).

## Discussion

Adults aged ≥85, the “oldest-old,” represent the fastest-growing segment of the population and account for a meaningful share of hospitalizations (1). While other causes for elderly hospitalization are declining, admissions for UTIs and septicemia keep rising, reflecting BOO and catheter dependence alongside heightened vulnerability to infection associated with frailty-related immunosenescence (21). Management of benign prostatic obstruction (BPO) in the elderly continues to pose a challenge. Without surgical intervention, these men often progress to acute urinary retention (AUR), become catheter-dependent, and subsequently experience higher rates of UTI and reduced quality of life (22). Conversely, operative management in this population has historically been thought to have suboptimal outcomes, particularly during the pre- and early HoLEP era when TURP was predominant (23).

Against this clinical backdrop, we examined HoLEP across two extremes of the age spectrum. Our study is the first to focus on outcomes of HoLEP in the ≥85 years group and compare them with men ≤55 years. Our findings demonstrate that HoLEP is safe and effective across a wide age range, although age-related differences in comorbidity burden, functional recovery, and complication profiles were evident. These observations align with the review by Yilmaz et al. who noted that laser enucleation of the prostate is effective and safe in elderly (>65 years), resulting in good functional outcomes, low morbidity, and few perioperative complications (24).

The oldest-old cohort in our study exhibited significantly greater multimorbidity and a higher prevalence of frailty, aligning with prior reports indicating high comorbidity burden in elderly patients undergoing BPH surgery (4, 5, 24). HT, CVD, CHF and PVD were significantly more common in the ≥85-year group, and COPD was observed exclusively in this cohort. Similar higher frailty scores, ASA scores, higher incidence of CVD, and anticoagulation were noted by other authors in octogenarians (25). These findings reflect that frailty is associated with advanced age and may be more clinically actionable than chronological age alone when counseling the oldest-old. Incorporating a structured frailty assessment into the preoperative evaluation and risk stratification can strengthen shared decision-making.

We noted that younger patients exhibited significantly higher BMI compared to oldest-old group similar to Elsaqa M. et al. who noted that octogenarians had lower BMI compared with those <80 years (25). It has been reported that with each 0.05 increment increase in waist-to-hip ratio there is a 10% increased risk of total and severe BPH (26). We believe that obesity might play a role in BPH progression in these men, resulting in a larger prostate size comparable to that of older men. Expectedly, the prostate size was larger in oldest-old as compared to middle-aged men, although the difference was not statistically significant. Our findings align with many studies revealing a larger prostate in elderly reflecting a pattern of prostatic growth with age (10). Studies have shown that the average increase of prostate volume ranges from 1.6 to 2.5% per year (27).

In our cohort, operative times and length of stay were comparable in contrast with Piao et al., who noted higher operative time and a longer hospital stay in patients aged ≥80 years (17). We noted longer duration of postoperative catheterization in the elderly similar to Mmeje et al. (18) Functional outcomes improved meaningfully in both groups. Despite higher baseline PVR in the oldest-old, improvement in IPSS and PVR was comparable. Although 70% of the study group were catheter dependent, all patients in both groups achieved spontaneous voiding. This is consistent with prior findings of high catheter-free rates following laser enucleation in men ≥80 years (5). Conversely, Lotterstätter et al. reported that among patients ≥85 years undergoing TURP, 12.9% of them remained catheter-dependent and 0.6% died, underscoring the potential advantage of HoLEP in achieving catheter-free status with minimal mortality in this vulnerable population (11). In the younger cohort, although the prevalence of catheter dependence was significantly lower, nearly one-third of men ≤55 years were catheter-dependent preoperatively, which is remarkably high for this age group. This reflects a highly selected subgroup enriched for absolute indications such as recurrent urinary retention and catheter dependence, similar to Gildor et al.’s findings (19).

Consistent with existing literature, we noted that younger patients demonstrated greater improvement in Qmax at each follow-up interval, which reached statistical significance at 12 months (28). The more modest Qmax gains observed in the ≥85-year cohort align with evidence of bladder dysfunction related to longstanding bladder outlet obstruction and age-related detrusor dysfunction (6). Despite this, improvements in IPSS and PVR were comparable between groups, mirroring findings from Piao et al. and Rivera et al., who reported preserved symptomatic improvement across various age categories (17, 29).

Complication rates were generally low. However, it is worth emphasizing that the elderly exhibited almost a 2-fold increase in minor CD ≤ 2 (60% vs. 35.4%) and a 3-fold increase in major CD ≥ 3 complications (10% vs. 3.2%) and three patients in the study group required blood transfusion with subsequent ICU admission. Two were markedly anemic preoperatively despite repeated transfusions; HoLEP was undertaken as a salvage procedure in these medically fragile patients, and they were monitored in the ICU. The third patient developed significant postoperative hematuria with ongoing bleeding and a rapid hemoglobin decline despite transfusion, necessitating ICU observation. While older patients in our study exhibited higher rates of transfusion, the difference did not reach statistical significance. Our findings align with Mmeje et al. who noted 11% incidence of blood transfusion in octogenarians (18). Results from the American College of Surgeons National Surgical Quality Improvement Program (ACS-NSQIP) database from 2011 to 2020 also noted higher transfusion requirements after HoLEP in octogenarians when compared with non-octogenarians (4). The higher proportion of anticoagulation might explain the higher incidence of transfusion in the elderly. UTIs requiring antibiotic therapy occurred only in the ≥85-year cohort and were likely related to catheter dependence, and frailty-related immune dysregulation as suggested by Woodford et al. (21) Transient incontinence was more common in the elderly, aligning with Savin et al., who reported higher TUI rates in patients ≥80 years (16). Notably, the higher rate of minor complications (Clavien–Dindo ≤2) observed in the oldest-old cohort was primarily driven by urinary tract infections, transient urinary incontinence, and transfusion requirements. Although classified as “minor,” these events may adversely affect recovery trajectories and contribute to increased health care utilization. These findings underscore the importance of incorporating age-specific risk profiles into preoperative counseling and postoperative planning, including proactive surveillance, early infection management, continence support, and caregiver planning.

Prior studies consistently reported higher iPCa detection in older men undergoing HoLEP or TURP largely due to age-related increases in PSA screening discontinuation, prostate volume growth, and occult disease burden (5, 17). Our study also demonstrated higher detection of iPCa in elderly patients as documented in literature (30). Additionally, most of the iPCa were GG1.

This analysis is limited by its retrospective design, modest sample sizes within the extreme age groups, and variability in follow-up adherence at later time points. Additionally, functional outcomes were analyzed using cross-sectional comparisons at each follow-up visit; we did not perform longitudinal modelling Therefore, attrition at later time points, particularly among men ≥85 years, may introduce a bias limiting the precision of 12-month estimates. Despite these limitations, our study adds to the literature supporting HoLEP as a paradigm-shifting alternative due to its superior hemostatic profile and durable outcomes irrespective of prostate size or patient comorbidities. It not only supports the safety and effectiveness of HoLEP in the oldest-old but also reinforces the observation that younger individuals with shorter-standing obstruction tend to recover better and achieve greater Qmax. These findings suggest that delaying surgical intervention may limit functional recovery due to progressive, potentially irreversible bladder dysfunction while increasing the risk of complications. Although younger patients exhibited greater improvements in urinary flow, whether this reflects a causal benefit of earlier intervention remains uncertain and requires prospective validation.

## Conclusion

Our study suggests that HoLEP provides meaningful functional improvements in the oldest-old who were historically considered high-risk or suboptimal surgical candidates. By directly comparing individuals at the extremes of age, greater recovery in Qmax is observed in younger patients. It is uncertain whether this finding reflects a causal benefit of earlier intervention, underscoring the need for prospective validation.

## Data Availability

The data analyzed in this study is subject to the following licenses/restrictions: the raw data supporting the conclusions of this article will be made available by the authors, without undue reservation, upon reasonable request and after approval by the institutional review board. Requests to access these datasets should be directed to Hemendra N. Shah, hns35@med.miami.edu.
